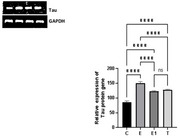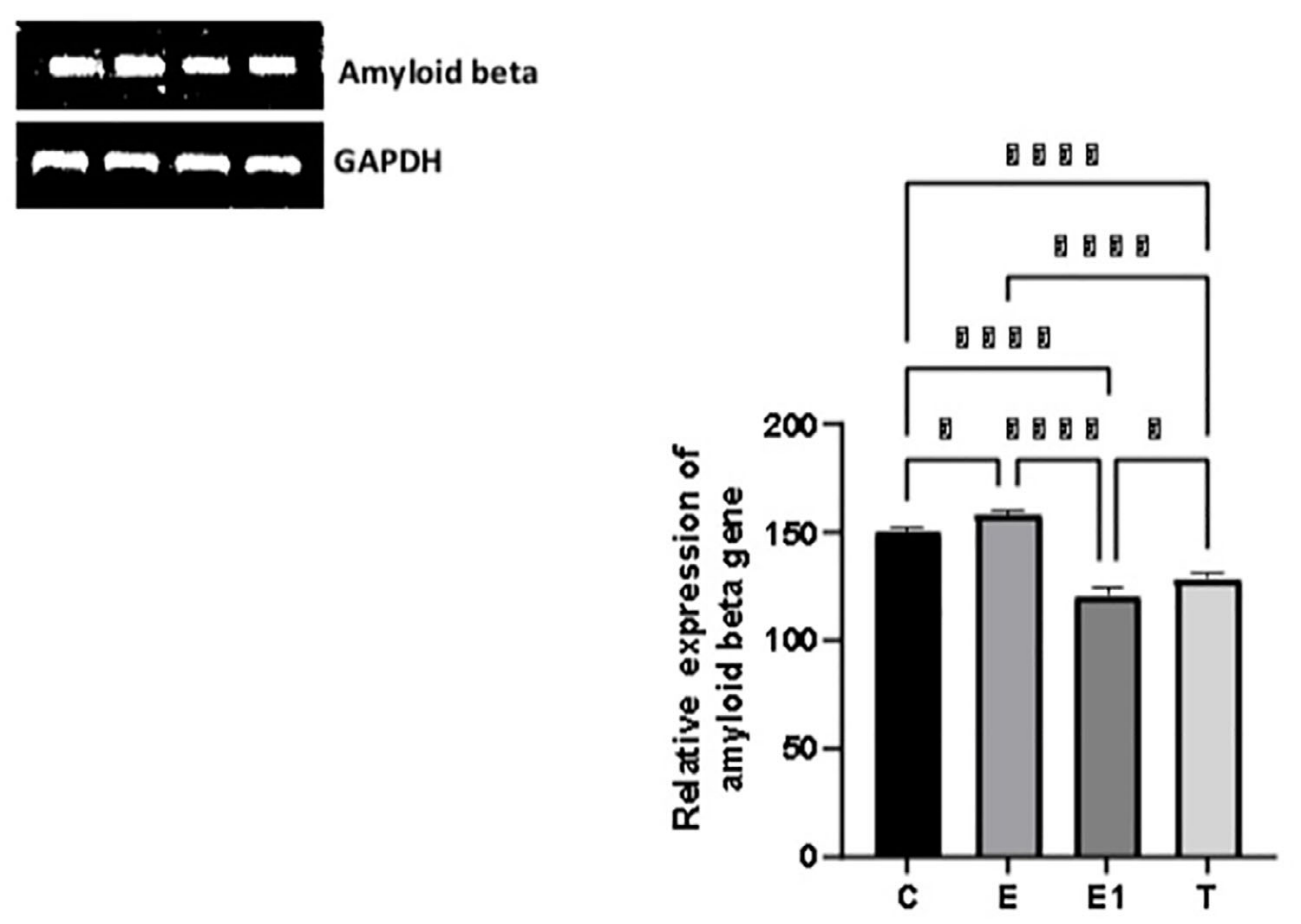# Inhibited FSH Alterations On Alzheimer'S Disease Rodent Models: Focus on tau and Amyloid Beta Expression

**DOI:** 10.1002/alz70856_098176

**Published:** 2025-12-24

**Authors:** Chinyem Nkemjika Ighodaro, Abigail Meno Akhigbemen

**Affiliations:** ^1^ University of Benin, Benin city, Edo, Nigeria

## Abstract

**Background:**

Reproductive hormones have been implicated to play a role in the occurrence of Alzheimer's disease (AD); a neurodegenerative disorder with multifactorial aspects which include genetic, hormonal and environmental factors. Studies have shown that a rise in Luteinizing hormone (LH) leads to increased AD prevalence in menopausal women. During menarche, LH and Follicle stimulating hormone (FSH) work synergistically to regulate menstrual cycles. Recent studies have suggested the early initiation of hormonal treatment during menarche to reduce the onset in AD at menopausal age. Rodents have an estrous cycle similar to the menstrual phase in humans. This study was aimed at investigating the expression of tau protein and amyloid beta in the hippocampi of rodents in their estrous phase by inhibiting FSH levels.

**Method:**

A total of 40 Albino wistar rats (250‐ 300 grams) were used for this study. The rats were randomly assigned to 4 groups with a total of 10 rats per group. Group C served as control (water and feed ad libitum) Group T (AlCl_3_ only 100 mg/ kg body weight for 25 days); Group E1 (AlCl_3_100 mg/kg body weight for 25 days and post treated with Estradiol 3 mg/kg for 21 days); Group E (Estradiol only 3 mg/kg body weight for 21 days). AD was confirmed in groups 2 and 3 with neuro‐behavioral assessment standards. Thereafter, the hippocampi were exercised and homogenized for the analysis of tau protein and amyloid beta gene expression using reverse‐transcriptase polymerase chain reaction (RT‐PCR)

**Result:**

Inhibited FSH statistically significantly decreased the expression of Tau in the rats induced with AD when compared with control group (*p* <0.05), however a statistically significant increase in amyloid beta expression was observed in the group induced with AD when compared to control (*p* <0.05).

**Conclusion:**

Our results suggest that inhibited FSH gave rise to a down regulated Tau protein expression, on the contrary an upregulation in the expression of amyloid‐beta was observed. These findings raises our interests on reproductive hormonal interplay in AD, more studies are needed to understand the role of follicle stimulating hormone in menarche which may impact on the occurrence of AD in women.